# Identifying Ashkenazi Jewish *BRCA1/2* founder variants in individuals who do not self-report Jewish ancestry

**DOI:** 10.1038/s41598-020-63466-x

**Published:** 2020-05-06

**Authors:** Ruth I. Tennen, Sarah B. Laskey, Bertram L. Koelsch, Matthew H. McIntyre, Joyce Y. Tung

**Affiliations:** 23andMe, Inc., 223 N Mathilda Ave, Sunnyvale, CA 94086 USA

**Keywords:** Cancer prevention, Cancer genetics, Genetic testing, Cancer genetics, Cancer prevention

## Abstract

Current guidelines recommend *BRCA1* and *BRCA2* genetic testing for individuals with a personal or family history of certain cancers. Three *BRCA1/2* founder variants — 185delAG (c.68_69delAG), 5382insC (c.5266dupC), and 6174delT (c.5946delT) — are common in the Ashkenazi Jewish population. We characterized a cohort of more than 2,800 research participants in the 23andMe database who carry one or more of the three Ashkenazi Jewish founder variants, evaluating two characteristics that are typically used to recommend individuals for *BRCA* testing: self-reported Jewish ancestry and family history of breast, ovarian, prostate, or pancreatic cancer. Of the 1,967 carriers who provided self-reported ancestry information, 21% did not self-report Jewish ancestry; of these individuals, more than half (62%) do have detectable Ashkenazi Jewish genetic ancestry. In addition, of the 343 carriers who provided both ancestry and family history information, 44% did not have a first-degree family history of a *BRCA*-related cancer and, in the absence of a personal history of cancer, would therefore be unlikely to qualify for clinical genetic testing. These findings may help inform the discussion around broader access to *BRCA* genetic testing.

## Introduction

Pathogenic variants in the *BRCA1* and *BRCA2* genes are linked to an increased risk for female breast and ovarian cancer (including early-onset breast cancer), male breast cancer, prostate cancer, pancreatic cancer, and certain other cancers^1^. These variants are highly penetrant: Women with a variant have a 45–85% chance of developing breast cancer and up to a 46% chance of developing ovarian cancer by age 70^2^. However, increased surveillance and prophylactic surgery (mastectomy and salpingo-oophorectomy) can greatly reduce the risk of breast and ovarian cancer in women carrying a *BRCA1* or *BRCA2* mutation^3^.

The prevalence of pathogenic *BRCA1* and *BRCA2* variants is estimated to be between 1 in 300 and 1 in 800 in the general population^1,2^. Among individuals of Ashkenazi Jewish descent, three *BRCA1/2* founder variants — 185delAG (c.68_69delAG), 5382insC (c.5266dupC), and 6174delT (c.5946delT) — are present at a frequency of ~1 in 40^1^.

Current U.S. guidelines limit *BRCA1*/2 genetic testing to individuals with a personal or family history of a relevant cancer, including early-onset breast cancer, multiple primary breast cancers, ovarian cancer, and certain other cancers^1,2,4^. In addition, Ashkenazi Jewish ancestry is sometimes used to recommend screening for individuals with a personal or family history of a single breast cancer at any age^1^. However, recent studies have found that about 50% of *BRCA* carriers have little or no family history of a relevant cancer^5–8^. These individuals would likely not qualify for clinical genetic testing unless they developed cancer themselves, representing a missed opportunity for cancer prevention. Because *BRCA* variants predispose to very high breast and ovarian cancer risks even among carriers without a family history^5,9^, these findings have spurred calls for broader access to *BRCA* genetic testing, among Ashkenazi Jews and in the general population^5–7,10,11^.

The 23andMe database provides an ethnically diverse group of genotyped individuals. We sought to characterize a cohort of individuals who carry one or more of the three Ashkenazi Jewish founder variants as related to two characteristics that are typically used to recommend individuals for *BRCA* testing: self-reported Jewish ancestry and family history of breast, ovarian, prostate, or pancreatic cancer. We focused on these two characteristics because they can enable individuals to learn their *BRCA* status before developing cancer, thus providing opportunities for cancer prevention and/or early detection.

## Results

We identified 2,853 individuals who carry one or more of the three Ashkenazi Jewish *BRCA1/2* founder variants (Table 1).

**Table 1:** Demographics of 2,853 individuals carrying one or more of the three Ashkenazi Jewish *BRCA* founder variants.

Age (years)	Men	Women
Overall	1539	1314
18–30	205 (13.3%)	186 (14.2%)
31–50	517 (33.6%)	461 (35.1%)
51–70	550 (35.7%)	510 (38.8%)
71+	267 (17.3%)	157 (11.9%)

We first characterized the ethnic backgrounds of the carriers. The three variants in this study are most common in people of Ashkenazi Jewish descent; overall, 1 in 46 23andMe research participants with 85–100% Ashkenazi Jewish genetic ancestry had at least one of these variants, consistent with previous reports that the frequency of these variants in the Ashkenazi Jewish population is approximately 1 in 40^1^. However, among the 1,967 carriers who provided self-reported ancestry information, 21% did not report Jewish ancestry (Table 2). Participation in this study was not restricted by ethnicity or country of residence for otherwise eligible participants; additional self-reported ethnicity information is provided in Supplementary Table 1.

**Table 2:** Self-reported Jewish ancestry in 1,967 *BRCA* carriers.

		Self-reported Jewish ancestry	Did not self-report Jewish ancestry
Overall	1967	1552 (78.9%, of 1967)	415 (21.1%, of 1967)
- *BRCA1* 185delAG	748	625 (83.6%, of 748)	123 (16.4%, of 748)
- *BRCA1* 5382insC	415	192 (46.3%, of 415)	223 (53.7%, of 415)
- *BRCA2* 6174delT	811	741 (91.4%, of 811)	70 (8.6%, of 811)

1,967 of 2,853 carriers in the cohort provided self-reported ancestry information. The total number of *BRCA* variants detected exceeds the number of carriers because a small number of participants carry both a *BRCA1* and a *BRCA2* variant.

**Figure 1:**
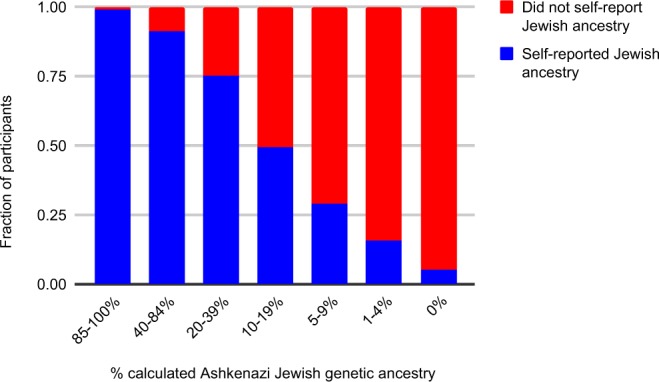
Self-reported Jewish ancestry vs. estimated Ashkenazi Jewish genetic ancestry in 1,967 *BRCA* carriers.

One possible explanation for the sizable fraction of carriers who did not report Jewish ancestry is that they were unaware of their Ashkenazi Jewish ancestry. To test this hypothesis, we explored the relationship between self-reported ancestry and genetic ancestry. As expected, individuals with a greater proportion of estimated Ashkenazi Jewish genetic ancestry were more likely to report Jewish ancestry (Table 3 and Fig. 1); fewer than half of individuals with less than 20% Ashkenazi Jewish genetic ancestry (roughly equivalent to one grandparent or great-grandparent who was Ashkenazi Jewish) reported Jewish ancestry. Furthermore, most (62%, 258 of 415) of the *BRCA* carriers who did not report Jewish ancestry did have at least 1% Ashkenazi Jewish genetic ancestry. However, a lack of knowledge of Ashkenazi Jewish ancestry could not fully account for the 21% of carriers who did not report Jewish ancestry, as 8.4% (166 of 1,967) of individuals carrying an Ashkenazi Jewish founder variant had no detectable Ashkenazi Jewish genetic ancestry. These data (Table 2, Table 3 and Supplementary Table 1) are consistent with previous reports that *BRCA1* 185delAG and 5382insC are also found in people of other ethnicities^12^. Nine individuals self-reported Jewish ancestry but had no detectable Ashkenazi Jewish genetic ancestry.

**Table 3:** Self-reported Jewish ancestry vs. estimated Ashkenazi Jewish genetic ancestry in 1,967 *BRCA* carriers.

Likely last fully Ashkenazi Jewish ancestor	% calculated Ashkenazi Jewish genetic ancestry	Self-reported Jewish ancestry	Did not self-report Jewish ancestry
Self	85–100%	972	10
1 parent or 2–3 grandparents	40–84%	419	41
1 grandparent	20–39%	88	29
1 grandparent or great-grandparent	10–19%	33	34
1 great-grandparent	5–9%	7	17
More distant than great-grandparent	1–4%	24	127
No detectable Ashkenazi Jewish ancestry	0%	9	157

The primary criterion for *BRCA* genetic testing is a personal or family history of breast, ovarian, or certain other cancers (including prostate and pancreatic cancer)^1^. We therefore assessed whether the carriers in our cohort had a family history of cancer. 393 carriers provided family history information.

Among participants who reported Jewish ancestry, 41% reported no first-degree family history of a *BRCA*-related cancer (Table 4). Similarly, 54% of participants who did not report Jewish ancestry reported no first-degree family history of cancer. Although our family history data differ substantially from guidelines used to determine genetic testing eligibility (which often include age of diagnosis and more than one cancer of certain types), these data are consistent with previous reports that about 50% of *BRCA* carriers would not be eligible for genetic testing based on family history alone^5–8^.

**Table 4:** Self-reported first-degree family history of cancer in 393 *BRCA* carriers.

	Total number who provided family history information	Reported a family history of breast or ovarian cancer	Reported a family history of prostate or pancreatic cancer	Reported no family history of breast, ovarian, prostate, or pancreatic cancer
Self-reported Jewish ancestry	310	139 (45%)	71 (23%)	127 (41%)
Did not self-report Jewish ancestry	83	33 (40%)	9 (11%)	45 (54%)
Overall	393	172 (44%)	80 (20%)	172 (44%)

Percentages in each row may not sum to 100% because some participants reported a family history of more than one type of cancer.

## Discussion

In this study, we describe a cohort of approximately 2,800 individuals identified through direct-to-consumer genetic testing who carry one or more of the three Ashkenazi Jewish *BRCA1/2* founder variants. Among eligible participants, this cohort was not restricted by genetic ancestry, self-reported ethnicity, or country of residence. In characterizing the ancestry and family cancer histories of these individuals, we made two key observations.

First, we found that a sizable proportion (21%) of carriers do not self-report Jewish ancestry. Of these individuals, more than half (62%) do have detectable Ashkenazi Jewish genetic ancestry, although frequently in very low percentages (Table 3 and Fig. 1). Interestingly, 8% of carriers have no detectable Ashkenazi Jewish genetic ancestry, consistent with reports that *BRCA1* 185delAG and 5382insC are found in women of other ethnicities who are referred for clinical genetic testing^12^. One potential caveat is that the check-all-that-apply survey question format used to ascertain self-reported Jewish ancestry (“Do any of the following cultural group labels describe your ancestry? Please check all that apply.”) may mis-classify some individuals relative to a forced-choice format (e.g., “Do you have any Jewish ancestry?”)^13^. Thus, it is possible that we may be overestimating the fraction of carriers who do not know they have Jewish ancestry.

Second, we observed that nearly half of individuals carrying an Ashkenazi Jewish *BRCA* variant have no first-degree family history of a *BRCA*-related cancer and, in the absence of a personal cancer history, would therefore be unlikely to qualify for clinical genetic testing. This percentage is consistent with published reports that about 50% of *BRCA* carriers lack a strong family history of cancer^5–8^. Based on these findings, many individuals identified in our study likely would not have learned their *BRCA* status through traditional clinical testing; indeed, a recent study suggests that more than 80% of individuals with a pathogenic *BRCA* variant do not know they have one^8^.

One strength of this study is that we were able to analyze data from a large population not restricted by genetic ancestry, self-reported ethnicity, or prior personal or family history of cancer. However, there are likely to be differences between the 23andMe customers who consented to participate in this research and the general U.S. population that could impact the generalizability of our results, including education level, income, ethnicity, and knowledge of/interest in genetics. In addition, while we have no reason to believe that people with a family history of cancer are more likely to become 23andMe research participants, we cannot exclude this possibility.

Other limitations of this study include a potential ascertainment bias related to family cancer history within the 23andMe database itself, as individuals with such histories may be more likely to answer questions about family cancer history. In addition, due to the limited depth of our family history survey, we defined family history as having a first-degree relative with breast, ovarian, prostate, or pancreatic cancer; clinical testing criteria are typically stricter, requiring an early age of diagnosis and/or more than one affected family member. Together, these two points suggest that our estimate of the fraction of individuals who would be ineligible for testing based on family history alone under existing screening guidelines is likely lower than the true fraction. Finally, because not all individuals in this study provided ancestry and family history information, the number of individuals included in some analyses is fairly small.

Our data suggest that a sizable fraction of individuals with detectable Ashkenazi Jewish genetic ancestry are unaware of that ancestry. This phenomenon is likely not unique to Ashkenazi Jewish ancestry. In addition to *BRCA*-related cancers, many other conditions are more common in specific ancestral groups, including Tay-Sachs disease, Canavan disease, and Gaucher disease type 1 in Ashkenazi Jews; sickle cell anemia in individuals with African ancestry; and beta-thalassemia in individuals with Mediterranean and certain other ancestries. For individuals who are unaware of their genetic ancestry, perceived risk for diseases could thus differ substantially from actual risk, which could lead to missed opportunities for genetic screening, prevention, and early intervention.

In recent years, several groups have called for broader access to *BRCA* genetic testing among Ashkenazi Jews and among women in the general population, which could enable women and men with a *BRCA* variant to learn their status, take steps to reduce their cancer risk, and encourage cascade testing of close family members^5–7,10,11^. Among Ashkenazi Jews, where testing for the three founder variants can identify most *BRCA* carriers, population-wide screening is cost-effective or even cost-saving^14^; in other ethnicities, more comprehensive genetic testing would be required to identify most individuals carrying a *BRCA* variant, but depending on the source of testing, this may also be cost-effective^15^. Our data may help inform the discussion around this growing call for expanded *BRCA* testing.

## Methods

Participants were drawn from the customer base of 23andMe. All participants provided informed consent and answered surveys online according to a research protocol approved by Ethical and Independent Review Services, an external AAHRPP-accredited institutional review board. Data on ancestry and family cancer history were collected by self-report via online surveys; see Supplementary Information for survey questions. All consented 23andMe research participants 18 years or older and genotyped on one of the two arrays described below were eligible for the study, regardless of self-reported or genetic ancestry. Analyses were run on phenotypic data collected before October 10, 2017. All research was performed in accordance with relevant guidelines and regulations.

DNA extraction and genotyping were performed on saliva samples by CLIA-certified and CAP-accredited clinical laboratories of Laboratory Corporation of America. Samples were genotyped on one of two custom Illumina genotyping arrays^16^: the OmniExpress+ Bead chip (V3) or a fully custom array (V4). The three variants included in this study (185delAG, 5382insC, and 6174delT) are returned to 23andMe customers as part of 23andMe’s Health + Ancestry Service and have been analytically validated on the most recent versions of our genotyping chip.

Proportions of Ashkenazi Jewish genetic ancestry were estimated via an analysis of local genetic ancestry as described previously^17^. To account for imprecision in genetic ancestry estimates, we characterized estimates of Ashkenazi Jewish genetic ancestry <1% as “not detectable”.

## Supplementary information

Supplementary Information.

## Data Availability

All data generated or analyzed during this study are included in this published article.
