# Supplementary material for: Identifying Ashkenazi Jewish BRCA1/2 founder variants in individuals who do not self-report Jewish ancestry
Source: Sci Rep. 2020 May 6;10:7669. doi: 10.1038/s41598-020-63466-x (PMC7203114; doi:10.1038/s41598-020-63466-x)
Supplement: Supplementary file 1 — Supplementary Information. [file 41598_2020_63466_MOESM1_ESM.pdf]

Supplementary Information

Identifying Ashkenazi Jewish *BRCA1/2* founder variants in individuals who do not self-report Jewish ancestry

Ruth I. Tennen[1], Sarah B. Laskey[1], Bertram L. Koelsch[1], Matthew H. McIntyre[1], Joyce Y. Tung[1]*

[1]23andMe, Inc., 223 N Mathilda Ave, Sunnyvale, CA, 94086 USA

*Corresponding author: joyce@23andme.com

Supplementary Table 1. Self-reported race / ethnicity of a subset of *BRCA* carriers

	185delAG	5382insC	6174delT
White; White/Native American	648	384	752
White, Hispanic; Native American/White, Hispanic	37	≤10	≤10
Middle Eastern/White; Middle Eastern	25	≤10	25
White/East Asian; South Asian	≤10	0	≤10
Black; Black/White	≤10	≤10	≤10
White/Other; Other, Hispanic; Other	31	11	27

Data from different ethnicities is grouped to protect the privacy of research participants. For the same reason, counts between 1 and 10 are elided. For all ethnicities included in the table, at least one study participant is a carrier of one or more of the three *BRCA1/2* variants listed.

Survey questions

Jewish ancestry

Do any of the following cultural group labels describe your ancestry? Please check all that apply.

[ ] Jewish
[ ] French Canadian
[ ] Cajun
[ ] Mennonite
[ ] Amish
[ ] Turkish
[ ] I'm not sure
[ ] None of the Above

Except for "I'm not sure" and "None of the above," the order of answer options was randomized for each participant. Participants who answered "Jewish" were counted as self-reporting Jewish ancestry; participants who gave any answer(s) other than "Jewish" were counted as not self-reporting Jewish ancestry; and participants who did not answer the question were not counted.

Race / ethnicity

Are you Hispanic or Latino?

( )Yes
( )No
( )I'm not sure

Participants who answered "Yes" were counted as self-reporting as Hispanic; participants who answered "No" were counted as not self-reporting as Hispanic; and participants who answered "I'm not sure" were not counted.

What is your race / ethnicity? Please check all that apply.

[ ] American Indian or Alaska Native
[ ] Asian
[ ] Middle Eastern
[ ] Native Hawaiian or Other Pacific Islander
[ ] Black, African, or African American
[ ] White or European
[ ] Other
[ ] I'm not sure

Except for "Other" and I'm not sure," the order of answer options was randomized for each participant. Participants who answered "I'm not sure" were not counted.

[For customers who selected "Asian" in the question above:]

What best describes your Asian ancestry? Please check all that apply.

[ ] Southeast Asian (e.g. Thai, Vietnamese, Indonesian, Filipino)
[ ] South Asian (e.g. Indian, Pakistani, Bangladeshi)
[ ] East Asian (e.g. Chinese, Japanese, Korean)

[ ] Central Asian (e.g. Kazakh, Uzbek, Tajik)
[ ] Other
[ ] I'm not sure

Except for "Other" and I'm not sure," the order of answer options was randomized for each participant.

Family cancer history

Participants provided information about family history of cancer in one of three surveys, which included slightly different versions of the family history questions. Functionally, there were two versions of the family history questions (described below). Version 1 was in one survey while Version 2 was in two surveys. Relevant questions from each survey are excerpted below.

Version 1

Is your family's history of cancer completely unknown to you for some reason, such as being adopted?

( ) Yes
( ) No

Please indicate below whether your biological parents or grandparents have ever had cancer. [Grandparental history was not used.]

Your mother

( )Yes
( )No
( )I'm not sure

Your father

( )Yes
( )No
( )I'm not sure

Have any of your other biological relatives, listed below, had cancer?

Your child(ren)

( )Yes
( )No
( )Not applicable
( )I'm not sure

Your sister(s)

( )Yes
( )No
( )Not applicable
( )I'm not sure

Your brother(s)

( )Yes
( )No
( )Not applicable
( )I'm not sure

{Where <female relative> could be “mother” or “sister”}
What type(s) of cancer was your <female relative> diagnosed with? Please check all that apply.
[ ] Adrenal gland cancer
[ ] Anal cancer
[ ] Bladder cancer
[ ] Biliary tract cancer
[ ] Brain cancer
[ ] Breast cancer
[ ] Cervical cancer
[ ] Colon/colorectal cancer
[ ] Endometrial or uterine cancer
[ ] Esophageal cancer
[ ] Gallbladder cancer
[ ] Hodgkin's lymphoma
[ ] Kidney/renal cancer
[ ] Leukemia, any type
[ ] Liver/bile ducts (hepatobiliary) cancer
[ ] Lung cancer
[ ] Melanoma
[ ] Mouth (oral) cancer
[ ] Non-Hodgkin's lymphoma
[ ] Pancreatic cancer
[ ] Ovarian cancer
[ ] Salivary gland cancer
[ ] Sarcoma, any type
[ ] Skin cancer (not melanoma)
[ ] Small intestine/duodenal cancer
[ ] Stomach/gastric cancer
[ ] Throat cancer
[ ] Thyroid cancer
[ ] Ureter/renal pelvis cancer
[ ] Vaginal cancer
[ ] Vulval cancer
[ ] Another type of cancer: _______________
[ ] I'm not sure

{Where <male relative> could be “father” or “brother”}
What type(s) of cancer was your <male relative> diagnosed with? Please check all that apply.
[ ] Adrenal gland cancer

[ ] Anal cancer
[ ] Bladder cancer
[ ] Biliary tract cancer
[ ] Brain cancer
[ ] Breast cancer
[ ] Colon/colorectal cancer
[ ] Esophageal cancer
[ ] Gallbladder cancer
[ ] Hodgkin's lymphoma
[ ] Kidney/renal cancer
[ ] Leukemia, any type
[ ] Liver/bile ducts (hepatobiliary) cancer
[ ] Lung cancer
[ ] Melanoma
[ ] Mouth (oral) cancer
[ ] Non-Hodgkin's lymphoma
[ ] Pancreatic cancer
[ ] Prostate cancer
[ ] Salivary gland cancer
[ ] Sarcoma, any type
[ ] Skin cancer (not melanoma)
[ ] Small intestine/duodenal cancer
[ ] Stomach/gastric cancer
[ ] Testicular cancer
[ ] Throat cancer
[ ] Thyroid cancer
[ ] Ureter/renal pelvis cancer
[ ] Another type of cancer: ______________
[ ] I'm not sure

What type(s) of cancer was your child(ren) diagnosed with? Please check all that apply.
[ ] Adrenal gland cancer
[ ] Anal cancer
[ ] Bladder cancer
[ ] Biliary tract cancer
[ ] Brain cancer
[ ] Breast cancer
[ ] Cervical cancer
[ ] Colon/colorectal cancer
[ ] Endometrial or uterine cancer
[ ] Esophageal cancer
[ ] Gallbladder cancer
[ ] Hodgkin's lymphoma
[ ] Kidney/renal cancer

[ ] Leukemia, any type
[ ] Liver/bile ducts (hepatobiliary) cancer
[ ] Lung cancer
[ ] Melanoma
[ ] Mouth (oral) cancer
[ ] Non-Hodgkin's lymphoma
[ ] Pancreatic cancer
[ ] Prostate cancer
[ ] Ovarian cancer
[ ] Salivary gland cancer
[ ] Sarcoma, any type
[ ] Skin cancer (not melanoma)
[ ] Small intestine/duodenal cancer
[ ] Stomach/gastric cancer
[ ] Testicular cancer
[ ] Throat cancer
[ ] Thyroid cancer
[ ] Ureter/renal pelvis cancer
[ ] Vaginal cancer
[ ] Vulval cancer
[ ] Another type of cancer: _____________
[ ] I'm not sure

Have you ever been diagnosed with any of these hereditary cancer syndromes?
[ ] Cowden syndrome
[ ] Familial adenomatous polyposis
[ ] Hereditary breast and ovarian cancer syndrome (HBOC)
[ ] Li-Fraumeni syndrome
[ ] Lynch syndrome (hereditary non-polyposis colorectal cancer syndrome)
[ ] Multiple endocrine neoplasias
[ ] Von Hippel-Lindau Disease
[ ] I'm not sure
[ ] None of the above

Version 2
Please indicate below whether your biological parents, siblings, or children have ever had cancer.
Your mother
( )Yes
( )No
( )I'm not sure

Your father
( )Yes

( )No
( )I'm not sure

Your sibling(s)
( )Yes
( )No
( )I'm not sure

Your child(ren)
( )Yes
( )No
( )I'm not sure

How many biological siblings do you have? _____

How many biological children do you have? _____

What type(s) of cancer was your biological mother diagnosed with or treated for? Please select all that apply.
[ ] Bladder cancer
[ ] Brain cancer
[ ] Breast cancer
[ ] Colon/colorectal cancer
[ ] Endometrial or uterine cancer
[ ] Hodgkin's lymphoma
[ ] Kidney/renal cancer
[ ] Leukemia, any type
[ ] Liver cancer
[ ] Lung cancer
[ ] Myeloma
[ ] Non-Hodgkin's lymphoma
[ ] Ovarian cancer
[ ] Pancreatic cancer
[ ] Skin cancer
[ ] Stomach cancer
[ ] Thyroid cancer
[ ] Another type of cancer
[ ] I'm not sure

What type(s) of cancer was your biological father diagnosed with or treated for? Please select all that apply.
[ ] Bladder cancer
[ ] Brain cancer
[ ] Colon/colorectal cancer

[ ] Esophageal cancer
[ ] Hodgkin's lymphoma
[ ] Kidney/renal cancer
[ ] Leukemia, any type
[ ] Liver cancer
[ ] Lung cancer
[ ] Mouth (oral) cancer
[ ] Myeloma
[ ] Non-Hodgkin's lymphoma
[ ] Pancreatic cancer
[ ] Prostate cancer
[ ] Skin cancer
[ ] Stomach cancer
[ ] Thyroid cancer
[ ] Another type of cancer
[ ] I'm not sure

How many of your biological siblings have ever had cancer? _____

What type(s) of cancer was your biological sibling(s) diagnosed with or treated for? Please select all that apply.
[ ] Bladder cancer
[ ] Brain cancer
[ ] Breast cancer
[ ] Colon/colorectal cancer
[ ] Endometrial or uterine cancer
[ ] Esophageal cancer
[ ] Hodgkin's lymphoma
[ ] Kidney/renal cancer
[ ] Leukemia, any type
[ ] Liver cancer
[ ] Lung cancer
[ ] Mouth (oral) cancer
[ ] Myeloma
[ ] Non-Hodgkin's lymphoma
[ ] Ovarian cancer
[ ] Pancreatic cancer
[ ] Prostate cancer
[ ] Stomach cancer
[ ] Skin cancer
[ ] Thyroid cancer
[ ] Another type of cancer
[ ] I'm not sure

How many of your biological children have ever had cancer? _____

What type(s) of cancer was your biological child(ren) diagnosed with or treated for? Please select all that apply.

[ ] Bladder cancer
[ ] Brain cancer
[ ] Breast cancer
[ ] Colon/colorectal cancer
[ ] Endometrial or uterine cancer
[ ] Esophageal cancer
[ ] Hodgkin's lymphoma
[ ] Kidney/renal cancer
[ ] Leukemia, any type
[ ] Liver cancer
[ ] Lung cancer
[ ] Mouth (oral) cancer
[ ] Myeloma
[ ] Non-Hodgkin's lymphoma
[ ] Ovarian cancer
[ ] Pancreatic cancer
[ ] Prostate cancer
[ ] Stomach cancer
[ ] Skin cancer
[ ] Thyroid cancer
[ ] Another type of cancer
[ ] I'm not sure

Have any of your biological parents, siblings, or children ever been diagnosed with any of these hereditary cancer syndromes?

[ ] Cowden syndrome
[ ] Familial adenomatous polyposis
[ ] Hereditary breast and ovarian cancer syndrome (HBOC)
[ ] Li-Fraumeni syndrome
[ ] Lynch syndrome (hereditary non-polyposis colorectal cancer syndrome)
[ ] Multiple endocrine neoplasias
[ ] Von Hippel-Lindau Disease
[ ] I'm not sure
[ ] None of the above
